# Elucidating Potential Profibrotic Mechanisms of Emerging Biomarkers for Early Prognosis of Hepatic Fibrosis

**DOI:** 10.3390/ijms21134737

**Published:** 2020-07-03

**Authors:** Mishghan Zehra, James C. Curry, Sneha S. Pillai, Hari Vishal Lakhani, Cory E. Edwards, Komal Sodhi

**Affiliations:** Departments of Surgery and Biomedical Sciences, Marshall University Joan C. Edwards School of Medicine, Huntington, WV 25755, USA; humayun@marshall.edu (M.Z.); curry162@marshall.edu (J.C.C.); pillais@marshall.edu (S.S.P.); lakhani@marshall.edu (H.V.L.); coryedwards50@yahoo.com (C.E.E.)

**Keywords:** hepatic fibrosis, oxidative stress, miRNA, exosomes, cardiotonic steroids

## Abstract

Hepatic fibrosis has been associated with a series of pathophysiological processes causing excessive accumulation of extracellular matrix proteins. Several cellular processes and molecular mechanisms have been implicated in the diseased liver that augments fibrogenesis, fibrogenic cytokines and associated liver complications. Liver biopsy remains an essential diagnostic tool for histological evaluation of hepatic fibrosis to establish a prognosis. In addition to being invasive, this methodology presents with several limitations including poor cost-effectiveness, prolonged hospitalizations, and risks of peritoneal bleeding, while the clinical use of this method does not reveal underlying pathogenic mechanisms. Several alternate noninvasive diagnostic strategies have been developed, to determine the extent of hepatic fibrosis, including the use of direct and indirect biomarkers. Immediate diagnosis of hepatic fibrosis by noninvasive means would be more palatable than a biopsy and could assist clinicians in taking early interventions timely, avoiding fatal complications, and improving prognosis. Therefore, we sought to review some common biomarkers of liver fibrosis along with some emerging candidates, including the oxidative stress-mediated biomarkers, epigenetic and genetic markers, exosomes, and miRNAs that needs further evaluation and would have better sensitivity and specificity. We also aim to elucidate the potential role of cardiotonic steroids (CTS) and evaluate the pro-inflammatory and profibrotic effects of CTS in exacerbating hepatic fibrosis. By understanding the underlying pathogenic processes, the efficacy of these biomarkers could allow for early diagnosis and treatment of hepatic fibrosis in chronic liver diseases, once validated.

## 1. Introduction

Hepatic fibrosis, marked by profuse extracellular matrix (ECM) protein deposition in the liver [1,2], is a global health dilemma, contributing substantially to morbidity and mortality by affecting 100 million people worldwide [3,4,5]. The process of fibrosis entails significant alterations in the ECM production and remodeling [1,6]. Several factors collectively contribute to the disruption of intercellular contacts and interactions of ECM composition leading to excessive fibrosis in the liver [1,4,7,8,9,10]. Liver fibrosis can be a causative consequence of many chronic liver diseases [11], including chronic viral hepatitis B and C, non-alcoholic fatty liver disease (NAFLD), non-alcoholic steatohepatitis (NASH), alcoholic liver disease (ALD), autoimmune diseases, hemochromatosis [12,13,14,15], genetic disorders, excessive alcohol consumption, and metabolic disorders [16]. These underlying pathologies of liver fibrosis are also associated with metabolic syndrome and its components, such as obesity, type II diabetes, and insulin resistance [15,17,18]. Therefore, early assessment of liver fibrosis in a population with a high prevalence of etiologies associated with advanced fibrosis, as mentioned earlier, is essential [19,20,21].

Liver biopsy has long been considered as the gold standard for the histological evaluation and diagnosis of liver fibrosis and to establish a prognosis [22,23,24]. However, the clinical use of this method does not reveal the underlying pathogenetic mechanisms [22,23,25]. Apart from being invasive, the liver biopsy also have several limitations including, patient discomfort, considerable cost of treatment, prolonged hospitalizations and minimal risk of complications such as peritoneal bleeding. Furthermore, there may be sampling variation, poor sample quality, inter- and intra-observer variability and the possibility of error in small biopsy samples which may not reflect the fibrotic changes occurring in the entire liver [22,23,24,26]. Therefore, alternate strategies are required to supplant liver biopsy since it is not practical and affordable to biopsy each patient.

These limitations of liver biopsy that present a wide range of complications have led to the development of alternative noninvasive diagnostic strategies to estimate the extent of hepatic fibrosis [22,23,24]. One such approach is to evaluate hepatic biomarkers that are primarily categorized as direct, indirect, and combinatory markers [26]. These markers have been incorporated into the routine clinical care of patients with liver fibrosis and other liver diseases [27]. This dynamic noninvasive modality is affordable and can track disease progression and regression [26], with minimal complications and sampling errors, as seen with liver biopsy [26]. However, the diagnostic biomarkers currently used by clinicians lack specificity and sensitivity and thus unable to predict the etiology and stages of fibrosis [22,28]. A detailed evaluation of the extent of fibrosis is imperative for disease diagnosis, follow-up, and evaluating therapeutic response. Immediate diagnosis of hepatic fibrosis by noninvasive means would be more palatable than a biopsy, which would help the physicians to decide whether the patient requires a referral to a liver biopsy or not. The biomarkers could assist clinicians in taking early interventions timely, avoiding fatal complications, and improving prognosis, which would be enormously valuable. This biomarker-guided approach would provide a possibility to augment the diagnostic performance by detecting at-risk patients rapidly and readily. Several initiatives are underway to advance the research on the discovery of clinically significant biomarker candidates for liver fibrosis that could help clinicians in identifying disease progression earlier to improve the prognosis and also to expand the knowledge about the underlying mechanisms in the progression of fibrosis.

Different biological pathways involved in the exacerbation of liver fibrosis has put forward a deluge of putative biomarkers that need further validation prior to use in clinical practice. Therefore, we sought to review some common biomarkers of liver fibrosis along with some emerging candidates, including the oxidative stress-mediated biomarkers, epigenetic and genetic markers, exosomes, and miRNAs that needs further evaluation and would have better sensitivity and specificity. Copious studies have also highlighted the role of CTS in evoking tissue fibrosis. However, their role in liver fibrosis needs further investigations that can provide a comprehensive understanding of CTS as a potential candidate biomarker in elaborating liver fibrosis. Therefore, we also intend to evaluate the pro-inflammatory and profibrotic effects of CTS and the mechanism by which it does so.

## 2. Oxidative Stress Mediated Biomarkers

The liver has a high metabolic and synthetic activity; therefore, it is more susceptible to oxidative stress and inflammation produced by various enzymes and cells [29,30,31]. Reactive oxygen species (ROS) and reactive nitrogen species (RNS) are produced by normal metabolic pathways in the hepatic mitochondria and are kept at low levels by antioxidant mechanisms [29,32]. These free radicals are required for normal cellular functions but lead to oxidative stress when elevated [30]. The cumulative line of evidence suggests that oxidative stress and ROS stimulates profibrogenic mediators and plays a pivotal role during the initial liver inflammatory phase and its progression to fibrosis [3,30,33]. Such oxidative microenvironment could augment proinflammatory cytokine expression including IL-1β, IL-18, TNF-α [34], IL-17, IL-20, IL-33, chemokines like MCP-1, CXCL10 [35], stimulation of toll like receptor (TLR)-mediated signaling pathways [36], and activation of redox transcription factors such as NF-κB and AP-1, that up-regulates several genes correlated to fibrosis [35,37]. This aggravated intracellular signaling cascade recruit other cytokine secreting immune cells to the site of oxidative stress that eventually leads to the onset of an oxidative stress-mediated inflammatory response [35,38]. Studies have highlighted the significance of these cytokines and chemokines as potential markers of hepatic fibrosis, and elevated levels of cytokines in patients with liver fibrosis have been reported [39]. Furthermore, NADPH oxidase NOX-generated ROS has been suggested to play a preeminent role in the pathogenesis of liver fibrosis by promoting myofibroblast activation [40]. Regardless of etiology, these ROS can directly activate the prime executors of fibrogenesis i-e, hepatic stellate cells (HSCs) to transform into myofibroblasts and produce ECM proteins and further enhance the cellular oxidative stress and inflammation [3,41]. Reflexively, this redox-sensitive cascade results in the onset and exasperation of redox-related progression of liver fibrosis [7,35].

Activation of inflammatory cells and a substantial infiltration of macrophages, lymphocytes, and eosinophils [33], represents a significant source of oxidative stress-related molecules that mediate inflammation-associated profibrogenic effects [33,42,43,44,45]. Pathogenesis of liver fibrosis induced by hepatic inflammation is initiated with the release of apoptotic bodies [44], cytokines, and growth factors (TGFβ1, TNF-α, EGF, IGF) [6] from injured hepatocytes. These apoptotic bodies along with the cytokines activate quiescent HSCs, T-cells and resident macrophage in the liver, Kupffer cells [6], which together promote proinflammatory and fibrogenic response by releasing ROS, cytokines, including IL-1β, TNF-α, IL-17 [46], IL-33, IL-13, and TGFβ1, chemokines such as CCL2, CCL5 [8,44,47,48,49,50]. Activated quiescent HSCs under the influence of major fibrogenic cytokine TGFβ1, platelet-derived growth factor (PDGF), and endothelial growth factor (EGF), are transformed into myofibroblasts that enhance the secretion and deposition of ECM proteins [48,51,52]. Moreover, activated Kupffer cells promote fibrogenesis by further eliciting the production of ROS, activation of HSCs, and upregulating chemokines and cytokines, including IL-6 [43], TGFβ1, TNF-α, IGF [6]. Collectively, there is a vast repertoire of the inflammatory response with an aberrant increase of proinflammatory cytokines, chemokines, growth factors, immune cells [6,8,52,53], that mediates the process of liver fibrogenesis by nurturing hepatocyte necrosis, apoptosis and prolongs the tissue injury by favoring the process of ECM production in the liver [44,45]. Hence, evaluating serum levels of these markers on routine laboratory tests could serve as an affordable diagnostic and follow-up alternative to other expensive diagnostic approaches in a population with insufficient medical resources.

The cellular response to chronic injurious stimuli and uncontrolled repair processes during fibrogenesis mediated by oxidative stress and inflammation also results in significant alterations associated with both quantity and composition of ECM [44,50]. This is further accompanied by damaged hepatocytes which causes alterations in hepatic enzymes, such as ALT, AST, ALP, γGT [24]. Moreover, activated HSCs also produce MMP-2 [5], MMP-9, and MMP-3 to recruit inflammatory cells and participates in the process of liver fibrogenesis [6]. Therefore, these parameters could be used in diagnosing, evaluating severity, monitoring therapy and also assessing the prognosis of liver fibrosis [22].

**Figure 1:**
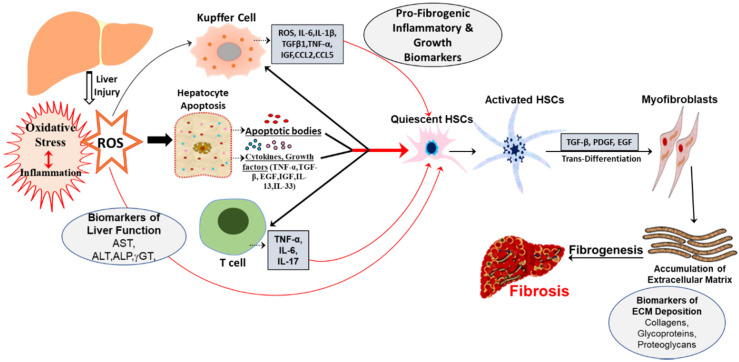
Schematic representation of pathogenic events and oxidative stress mediated biomarkers in hepatic fibrosis. Liver injury may be caused by multiples etiologies, including metabolic syndrome, NASH, NAFLD and ALD, that trigger oxidative stress and hepatic inflammation through different types of cells. This results in the generation of ROS/RNS that, in turn, induce apoptosis with the release of cytokines, chemokines, growth factors. These factors collectively enhance the activation of HSCs that ultimately leads to fibrosis.

As can be surmised from existing studies, several functionally diverse biomolecules could be developed as biomarkers for hepatic fibrosis. Since oxidative stress and inflammation is a conjoint pathogeneic event (Figure 1) underlying fibrogenesis, identification of molecular alterations presents at initial stages of oxidative damage leading to inflammation would be of great help in its early recognition and in monitoring the evolution of the disease.

## 3. Genetic and Epigenetic Markers

The highly complex genetics of progression and inter individual differences in liver fibrosis is multifactorial and have been attributed to environmental, genetic and epigenetic factors [54,55]. Linkage analysis of phenotypes and genotypes identified major chromosomes and genetic polymorphisms that have significant impact on different histological stage of fibrosis and hepatic collagen contents. Several fibrogenic gene variants such as complement component 5 [56], chemoattractants such as the chemokine CXCL9 [57], chemokine receptor, CXCR3 [58] and metabolic enzymes like the triglyceride hydrolase adiponutrin (PNPLA3) [59] have been identified. Quantitative trait locus (QTL) analysis identified seven genomic loci influencing fibrosis phenotypes with genome wide significance on chromosomes 4, 5, 7, 12, and 17 [60]. Studies have also shown that the locus on chromosome 2 encodes complement factor C5 and small molecule inhibitors of the C5a receptor displayed antifibrotic effects in vivo [54]. The chromosome 15, designated hepatic fibrogenic gene 1 (Hfib1), significantly affected the stage of fibrosis and hepatic collagen contents [61]. The polymorphisms in CXCL9 (monokine induced by interferon (IFN) γ), CXCL10 (IFN γ-inducible protein 10 (IP-10)), and CXCL11 (interferon-inducible T cell α chemoattractant; I-TAC) have been investigated in association with hepatic fibrosis [62,63]. The epigenetic mechanisms including DNA methylation, histone post translational modifications and non-coding RNAs are also involved in orchestrating many aspects of liver fibrogenesis such as chromatin structure, modification and initiation of transcription [64]. The epigenetic modulations on the peroxisome proliferator-activated receptor gamma (PPAR-γ) gene promoter and aberrant expression of a series of histones and chemokines in HSCs are reported to aggravate inflammation and oxidative stress, which in turn promotes differentiation of HSCs to myofibroblasts and augments the whole fibrogenesis process. The epigenetic modulations on matrix associated enzymes such as MMP and TIMP regulates the degradation process of ECM [65]. The detection of liver-derived epigenetic markers in the patient’s circulation can be used for the diagnosis and the epigenetic alterations on relevant genes can be used as a therapeutic target to reverse liver fibrosis.

## 4. MicroRNAs

The role of miRNAs as potential biomarker has been investigated by researchers, due to their implications in modulating several pathways. miRNAs are small single stranded molecules that are valuable as biomarkers as they are stable in bodily fluids. miRNA sequences are highly conserved among different species and their expression are specific to individual tissues or biological states [66]. A large group of miRNA families are involved in the activation of HSCs contributing to the development and progression of hepatic fibrosis. The miR-29 family, which include miR-29a, miR-29b, and miR-29c, act through various cellular signalling pathways such as NFκb pathway, TGFβ, and PI3K/AKT signaling for the progression of liver fibrosis [67]. It induces cell apoptosis by modulating phosphatidylinositol 3-kinase/AKT signaling pathway and regulates ECM accumulation [68]. The three members of the miR-34 family, including miR-34a, miR-34b and miR-34c, have pleiotropic roles in cell cycle, apoptosis, and cellular development. These miRNAs are upregulated in activated HSCs and plays a role in regulating the deposition of ECM proteins such as collagen, desmin, and αSMA [69], leading to altered expression of matrix metalloproteases (MMP) 1 and 2 [70]. miR-122 is one of the most abundant miRNAs in the liver that regulate several functions, including cell cycle, differentiation and apoptosis and are inversely linked to the severity of fibrosis [71,72]. The miR-15 family consists of six highly conserved miRNAs, including miR-15a/b, miR-16, miR-195, miR-497 and miR-322, which mainly regulate TGF-β signaling pathway [73]. miR15a, miR15b and miR-16 play pro-apoptosis roles in HSCs [74], while miR-16 is a pro-fibrotic factor and has a positive effect on TGF-β/Smad signaling pathway [75]. miR-195 plays an anti-fibrotic role in hepatic fibrosis by targeting cyclin E1 [76]. The miR-200 family has five members, including miR200a, miR-200b, miR-200c, miR-429 and miR-141 and suppresses β-catenin, a key factor of Wnt/β-catenin signaling pathway, which participates in liver remodelling and HSC activation [77], the miR-199 family has three members, including miR199a1, miR-199a2 and miR-199b and are well reported to possess pro-fibrogenic effect [78]. The miR-378 family includes eleven members, namely miR-378a, miR-378b, miR-378c, miR-378d1, miR-378d2, miR-378e, miR-378f, miR-378 g, miR-378 h, miR-378i and miR-378j, and participate in the suppression of activation of HSCs through directly targeting Gli3 [79]. miR-571 and miR-652 are linked to fibrogenic and inflammatory processes [80]. Thus, circulating microRNAs offer a biologically stable blood-based biomarker tool for detection of liver fibrosis.

## 5. Exosomes

**Figure 2:**
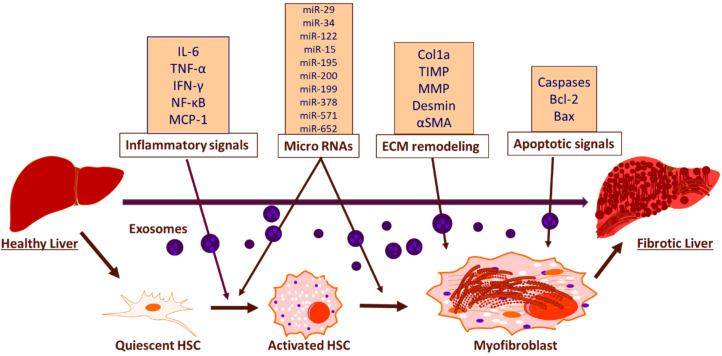
Schematic representation of exosomal function and biomarkers in inducing hepatic fibrosis. The exosomal mediated activation of HSCs induce hepatic fibrosis through the release of inflammatory biomarkers, miRNAs, biomarkers associated with ECM remodeling and apoptotic biomarkers.

The emerging field of exosome biology is currently exploring novel pathways of exosome mediated intercellular transfer of biologically active molecules that facilitate the development of liver fibrosis and other related liver pathologies. Recent studies have shown that exosomes function as mediators for intercellular transfer and contain all the necessary signals to induce fibrosis, such as macrophage activation and cytokine secretion, remodelling of ECM and modulation of HSCs [81] (Figure 2). The exosomal cargo present in blood and urine contain specific proteins, mRNAs and miRNAs, derived from liver and the differential expression of these exosomal cargo between healthy and diseased states can be considered as predictive biomarker for early detection and prognosis [82,83,84]. The cumulative line of evidence suggests decreased levels of miRNAs such as miR-34c, miR-151-3p, miR-483-5p, or miR-532-5p, in serum exosomes during fibrosis [85]. Studies have also shown that the activation of TLR3 in HSCs by exosomes derived from damaged hepatocytes exacerbates liver fibrosis by enhancing the production of chemokine (C-C motif) ligand 20 (CCL20), interleukin-17A (IL-17A), and exosomal miR-192, which significantly increases the expression of profibrotic markers in HSCs [86]. miR-214 can be considered as an anti-fibrotic marker as the level of miR-214 is increased in quiescent HSC -secreted exosomes as compared with activated HSC-released exosomes and it suppresses the expression of its direct target connective tissue growth factor (CTGF) and downregulates alpha-smooth muscle actin and collagen expression downstream of CTGF [87]. Reports show that damaged hepatocyte-derived exosomes containing P450s mediates the development of steatosis, increased fibronectin expression and hepatocyte apoptosis [88]. Furthermore, the activated HSC-derived exosomal CTGF is known to amplify fibrogenic signaling by intercellularly activating recipient HSCs [89]. Apart from that, CD81-enriched serum exosomes and an increase in the level of CD10 protein in urinary and serum exosomes have been shown as markers associated with inflammation and severity of fibrosis [84,90]. As the research advances, rising trend of approaches utilizing exosomes will open great source of diagnostic and prognostic molecular biomarkers for hepatic fibrosis.

## 6. Potential Role of Cardiotonic Steroids in Mediating Hepatic Fibrosis

CTS belongs to a group of volume-sensitive hormones [91] that seem to be both sufficient to alter transmembrane sodium transport in some cell types as well as to provoke a variety of cellular signals [92]. CTS can be structurally divided into two distinct categories, cardenolides that include ouabain and digoxin, and bufadienolides that includes telocinobufagin and marinobufagenin [91,93]. The synthesis and release of CTS are controlled by volume expansion and salt concentration, while it is regulated by the hormones of the hypothalamic-pituitary-adrenal axis [93,94]. Once released into the blood, CTS may have varying effects on different tissues throughout the body [95,96,97]. Differing concentrations of CTS allow for varying degrees of response [91,96,98]. Despite contentions, endogenous CTS represents an essential class of hormones with profound outcomes in health and disease [99,100]. Multiple lines of clinical and experimental evidence have suggested the prooxidant and profibrotic effects of these steroid hormones in different tissues [91,92,97,98,99,101,102,103]. Further studies have demonstrated that chronic exposure to nanomolar concentrations of CTS can result in hypertrophy and fibrosis in cardiac tissue, vasculature, dermal fibroblasts, and the kidneys [93,95,104,105], through various mechanisms, including the activation of collagen synthesis and other growth-related genes [94,96,105,106,107]. As CTS has shown links to fibrosis in various tissues, it is possible that it may be related to fibrosis elsewhere [97,105,107].

Reports on a number of endogenous CTS that are secreted in the body and regulated by multiple physiological stimuli have intensified research into their physiologic and pathophysiologic roles in the pathogenesis of different disease conditions [93,99]. CTS have also been found to play an essential role in the regulation of cholesterol biosynthesis [108,109]. Cholesterol is a crucial biomolecule that plays several structural and metabolic roles [110] and is a precursor for the synthesis of steroid hormones [111,112]. Moreover, cholesterol is a substrate for the biosynthesis of endogenous CTS as well [113,114]. The liver is the principal site for cholesterol homeostasis maintenance [115], and the accumulation of cholesterol in the liver can lead to pathological pictures such as fatty liver disease, NASH, NAFLD, and hepatic fibrosis [112,115]. Therefore, cholesterol homeostasis must be tightly regulated for proper cellular and systemic functions and to prevent over-accumulation and abnormal deposition [115,116]. Consequently, elevated levels of CTS could promote the accumulation of cholesterol in chronic liver disease and might result in substantial worsening and exacerbation of liver fibrosis by activating HSCs [117,118,119,120,121] and several redox-inflammatory pathways [112,122,123,124].

Substantive evidence has pointed to the role of CTS in Na/K-ATPase signaling [94,109,125,126,127], regulation of transcription factors [108,128,129] and modulation of hormone synthesis [130,131,132]. However, recent investigations on CTS have demonstrated an important role of digoxin and ouabain in eliciting cholesterol biosynthesis, which could be due to their sterol structure [108,109]. These cardenolides have shown to increase the synthesis of cholesterol without cellular toxicity at a concentration of 10nM to 1uM in HepG2 cells [108] by activating the mevalonate pathway and modulating the activity and expression of the rate regulatory enzyme of cholesterol biosynthesis, 3-hydroxy-3-methylglutaryl-CoA reductase (HMGCR) [108,109]. These findings imply that ouabain & digoxin by escalating cholesterol biosynthesis in the liver may have a role in aggravating the pathogenesis of chronic liver disease, including cholesterol-induced liver fibrosis.

**Figure 3:**
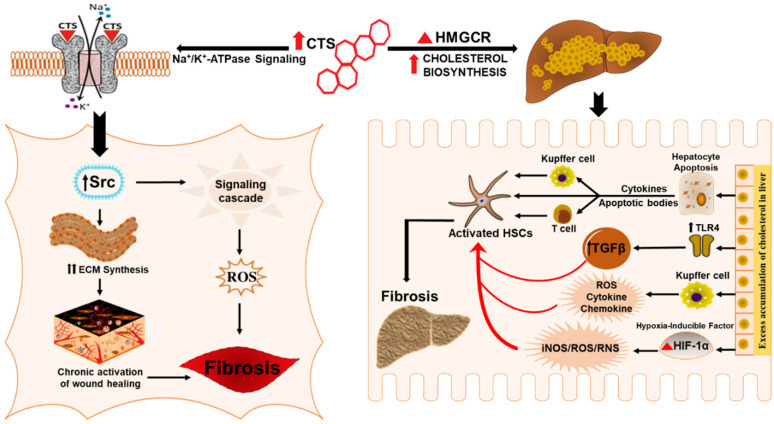
Schematic representation for the potential role of CTS in inducing hepatic fibrosis. CTS could potentiate liver fibrosis in chronic liver disease by inciting cholesterol biosynthesis, evoking wound healing effect and collagen synthesis through the CTS mediated activation of Na/K-ATPase signaling.

Emerging studies have suggested that altered hepatic cholesterol homeostasis & accumulation of oxidative products of cholesterol oxysterols and free cholesterol can affect the metabolic function and inflammatory status of the liver by precipitating hepatocyte injury and macrophage recruitment that in turn leads to the progression of liver fibrosis and its associated pathologies [112,133,134,135,136]. It appears that cardenolides by potentiating cholesterol synthesis and its associated pathways could have a role in the progression of liver fibrosis. Adding to that, the ambiguous effects of cholesterol also involve some signaling pathways that may promote liver fibrosis, including the over activation of SREBP-2 transcription factor [137], and NLRP3 inflammasome [138,139]. Also, excess cholesterol can induce the activation of kupffer cells that evoke inflammatory pathways and have been suggested to mediate hepatocyte lipotoxicity and promote the progression of hepatic fibrosis [140]. Furthermore, CTS could serve as an important link in cholesterol mediated liver fibrosis since an excess accumulation of cholesterol in hepatocytes results in hypoxic conditions and excessive generation of nitric oxide and mitochondrial ROS that aggravates the activity of oxygen-sensing transcription factor hypoxia-inducible factor HIF-1α, a significant regulator of liver fibrosis [112,124,141,142,143,144] and a potent activator of profibrotic redox-signaling molecule iNOS that is involved in the pathophysiology of cholesterol-induced liver fibrosis [124]. Moreover HSCs, regardless of etiology, are the prime contributors to liver fibrosis [145,146,147], and recent studies have shown that free cholesterol mediates the activation of HSCs by upregulating TLR4 protein and thereby rendering them susceptible to TGF-β induced activation in a vicious cycle of liver fibrosis [117,118,119,120,121]. Taken together, these findings suggest that CTS might play a contributory role in the progression of liver fibrosis by augmenting the biosynthesis of cholesterol (Figure 3) that, in turn, activates HSCs, redox, oxidant stress, inflammatory, and, HIF-1α pathways during chronic liver disease.

### 6.1. Cardiotonic Steroids in Wound Healing and Fibrosis

Chronic activation of wound healing is considered as the driving force of liver fibrogenesis [49,148,149,150,151] that has a worldwide clinical impact due to the high prevalence of CLD patients [149,150,151]. Wound healing is a complex biological event characterized by the synthesis of ECM proteins, e.g., collagen by massive differentiation of fibroblasts into myofibroblasts [151,152,153] whose activation is a critical process in the pathogenesis of tissue fibrosis [154]. Studies have reported the role of CTS in evoking a wound-healing effect by stimulating collagen synthesis (Figure 3) in human dermal fibroblast and cardiac fibroblast that involves signaling through the Na/K-ATPase pathway [102,104]. As the rate of collagen synthesis plays a major role in any tissue fibrosis [155,156] dysfunctions of this repair process can cause serious consequences, including hepatic fibrosis characterized by dysregulation of wound-healing response [157]. Therefore, CTS could potentially be exploited to exasperate the wound healing response in hepatic fibrosis by accelerating fibroblast proliferation, migration, and collagen production in the liver.

### 6.2. Cardiotonic Steroids Mediated Na/K-ATPase Signaling and Fibrosis

Several pathways and mechanisms have been described to unravel molecular mechanisms involved in the pathogenesis of liver fibrosis [15,48,158,159,160]. Elevated levels of circulating CTS potentially contribute to disease progression, such as in various chronic inflammatory conditions like obesity, hypertension, NASH, cardiovascular disease, and further stimulate the proinflammatory and profibrotic response [91,92,93,99,102,161,162,163,164]. Numerous studies have examined the role of the Na/K-ATPase ascribed by the scaffolding and signaling function in mediating organ fibrosis [91,101,165]. Many in vivo and in vitro studies have shown that CTS mediate signal transduction through Na/K-ATPase that induce signaling cascades, directly involved in the development of fibrosis in different tissues, including heart, kidney and vasculature [91,92,101,166,167]. The α-subunit of the Na/K-ATPase represents the specific receptor for CTS, and the signaling pathway involves the binding of the CTS to the caveolar Na/K-ATPase α-1 subunit [93,94,113,161,168,169]. We have also shown that oxidative stress and inflammation that plays a crucial role in the pathogenesis of fibrosis and ROS generated during this process can interact directly with the α1 subunit of Na/K-ATPase. This interaction, along with the binding of CTS, amplifies the signaling process via Src signaling cascade followed by the downstream modulation of the extracellular signal-regulated kinase (ERK1/2) [161]. CTS via Na/K-ATPase signaling could have implications in the progression of hepatic fibrosis through hypertrophic and fibrotic signaling pathways (Figure 3). Studies have also revealed the role of Na/K-ATPase signaling in maintaining cholesterol metabolism; consequently, CTS-mediated activation of Na/K-ATPase signaling in the liver could trigger the cholesterol-induced progression of liver fibrosis [170]. Morphological evidence showed increased lipid accumulation and hepatic fibrosis through CTS mediated activation of Na/K-ATPase signaling in high fat diet-fed mice. The study highlighted an increase in the inflammatory markers (MCP-1 and IL-6), marker of macrophage infiltration (F4/80) and an increase in fibrotic markers (Fibronectin, MMP-13 and MMP-9). Apart from inflammation and fibrosis, CTS mediated activation of Na/K-ATPase also altered the clinical features of NASH including insulin resistance, free fatty acid and lipid profile, which contributes to liver fibrosis. Together, these findings suggest that chronic stimulation of Na/K-ATPase signaling by CTS can result in the activation of the proinflammatory and profibrotic pathways in the liver, which could have significant implications in the pathogenesis of liver fibrosis.

## 7. Conclusions

Biomarkers are useful in debilitating consequences associated with liver fibrosis. The evaluation of emerging biomarkers that are involved in the pathogenesis of hepatic fibrosis, in the early assessment of fibrosis could have a significant influence on the patients and health care system. Given the potential burden caused by advanced liver fibrosis in a population with concomitant presence of metabolic syndrome, there is a considerable imperative to develop potential diagnostic modalities. Further extensive evaluations are required to circumscribe the predictive value of these biomarkers and to establish an accurate liver-specific biomarker panel for early diagnosis, management, mitigation of liver fibrosis, and to stratify patients for possible therapeutic interventions. Taken together, the observations described above indicated that oxidative stress and inflammation participates in a self-perpetuating cycle which, if not interrupted, can lead to progressive liver fibrosis. Furthermore, the genetic and epigenetic factors, along with highly sensitive miRNAs and exosomal proteins and bioactive mediators could serve as diagnostic biomarkers as well as therapeutic targets for hepatic fibrosis. Studies strongly implicate role of CTS in inflammation and fibrosis associated with chronic conditions. Multiple cellular and molecular signals have been investigated that contributes to liver fibrogenesis; many more have yet to be described. Among those signals, we tried to elucidate the potential role of CTS in triggering the progression of hepatic fibrosis by increasing the synthesis of cholesterol, eliciting a wound-healing response, and stimulating Na/K-ATPase signaling. To the best of our knowledge, there has not been any substantial attempt to explore a molecular connection between CTS and its role in aggravating liver fibrosis. Further in-depth studies are warranted and could shed light on the pathophysiological roles of CTS in the development of liver fibrosis, and that could be very meaningful to clarify their contribution in the amplification of this process.
